# Positive Network Assortativity of Influenza Vaccination at a High School: Implications for Outbreak Risk and Herd Immunity

**DOI:** 10.1371/journal.pone.0087042

**Published:** 2014-02-05

**Authors:** Victoria C. Barclay, Timo Smieszek, Jianping He, Guohong Cao, Jeanette J. Rainey, Hongjiang Gao, Amra Uzicanin, Marcel Salathé

**Affiliations:** 1 Center for Infectious Disease Dynamics, Department of Biology, The Pennsylvania State University, University Park, Pennsylvania, United States of America; 2 Department of Computer Science and Engineering, The Pennsylvania State University, University Park, Pennsylvania, United States of America; 3 Division of Global Migration and Quarantine, Centers for Disease Control and Prevention, Atlanta, Georgia, United States of America; Melbourne School of Population Health, Australia

## Abstract

Schools are known to play a significant role in the spread of influenza. High vaccination coverage can reduce infectious disease spread within schools and the wider community through vaccine-induced immunity in vaccinated individuals and through the indirect effects afforded by herd immunity. In general, herd immunity is greatest when vaccination coverage is highest, but clusters of unvaccinated individuals can reduce herd immunity. Here, we empirically assess the extent of such clustering by measuring whether vaccinated individuals are randomly distributed or demonstrate positive assortativity across a United States high school contact network. Using computational models based on these empirical measurements, we further assess the impact of assortativity on influenza disease dynamics. We found that the contact network was positively assortative with respect to influenza vaccination: unvaccinated individuals tended to be in contact more often with other unvaccinated individuals than with vaccinated individuals, and these effects were most pronounced when we analyzed contact data collected over multiple days. Of note, unvaccinated males contributed substantially more than unvaccinated females towards the measured positive vaccination assortativity. Influenza simulation models using a positively assortative network resulted in larger average outbreak size, and outbreaks were more likely, compared to an otherwise identical network where vaccinated individuals were not clustered. These findings highlight the importance of understanding and addressing heterogeneities in seasonal influenza vaccine uptake for prevention of large, protracted school-based outbreaks of influenza, in addition to continued efforts to increase overall vaccine coverage.

## Introduction

Influenza is an infectious disease affecting 5–20% of the population every year [1]. Schools are thought to play a major role in the spread of influenza into the community [2]–[4], and high transmission within schools is thought to be due to frequent, close contact between children [5], less acquired immunity in children [6], and more intense and prolonged shedding of the virus in children [7], [8]. Vaccines are a key tool to prevent influenza illness, and reduce disease spread [9]–[11]. Interrupting transmission of influenza in schools through high levels of vaccine-induced immunity among school-age children will protect unvaccinated individuals both in schools and in the wider community through the indirect protection offered by herd immunity [12]–[15]; that is, a reduction in transmission among both vaccinated and unvaccinated populations due to a high level of vaccine-induced immunity.

The effects of herd immunity are greatest when vaccination coverage reaches a critical threshold above which circulation of the respective pathogen will be interrupted. Conversely, average and maximal outbreak size will increase when vaccination coverage declines [16]. Lower vaccination coverage and diminished herd immunity have been blamed for the increased transmission and severity of outbreaks for a number of important diseases of public health concern [16], [17]. For example, this effect was observed in the Netherlands between 1999 and 2000 during a major measles outbreak, which primarily impacted members of a religious community that did not accept routine vaccination. This occurred despite national measles vaccination coverage >95%, the target threshold for measles control [18].

The assortativity coefficient, r, is used in network analysis to determine whether there is a tendency of nodes to associate with similar nodes with respect to a particular property (e.g., gender, ethnicity, vaccination status etc.,) [4], [19], [20]. If r>0, a network is said to be positively assortative for the property of interest [20]. Recent modeling studies on influenza transmission have begun to analyze the impact of non-random, positively assortative unvaccinated individuals in contact networks [21], [22]. These studies have shown that clusters of unvaccinated individuals can result in an increased likelihood of large disease outbreaks. Further investigation of whether vaccination is assortative becomes particularly important when we consider the spread of influenza in schools, as multiple generations of transmission through assorted unvaccinated students could increase the likelihood of community-wide outbreaks [13], [23], [24]. Such data will be necessary for models to accurately predict the impact of vaccination, the limits of herd immunity, and thus the potential size and duration of disease outbreaks.

In this study, we specifically measure empirically for the first time whether assortativity in seasonal influenza vaccination status exists within a close contact network at a United States (US) high school, and examine the potential significance of such vaccine assortativity on influenza outbreaks. To do this, we captured a high-resolution contact network using wireless sensor devices (“motes”) worn by members of a high school community across multiple days. We also distributed an online health survey, which asked members of the school to specifically indicate whether they had been vaccinated with the 2011/2012 influenza vaccine. Combined, these data allowed us to measure vaccination assortativity of the network and to run simulations of influenza disease outbreaks.

## Methods

### Ethics

To participate in the study, students less than 18 years old were asked to read an assent form that explained the study, and to provide written assent if they agreed to the study. Informed consent was not obtained from next of kin, caretakers or guardians on the behalf of the minors/children participants in the study because there was no greater than minimum risk to participants wearing the motes or completing the online health survey. Further, mote deployment and data retrieval followed previously published protocols in Salathé et al. 2010 [25]. The Pennsylvania State University IRB (IRB # 37640) and the CDC IRB authorization agreement approved the study protocol and related consent procedure for minors. Students over 18 years old, teachers, and staff were asked to read an informed consent form that explained the study and provide written consent if they agreed to the study.

The assent/informed consent forms also asked participants to provide their email address if they wanted to receive the online health survey. All participants received an extra form for their own records. Once the research team received the assent/informed consent forms, each participant was assigned a unique code number to protect participants' privacy. A project server stored code numbers and participants' information, and security measures were put in place to ensure the information was protected, including the framework Django, which has multiple levels of security installed by default. Only research team members named on the approved IRB applications had access to the list of code numbers and participants' information. After the data was collected, cleaned, and analyzed, the list linking code number to participants' information was destroyed. The Pennsylvania State University IRB (IRB # 37640) and the CDC IRB authorization agreement approved the study protocol with respect to data collection and storage.

### Data collection

Motes store close proximity records (CPRs), which are detection events for face-to-face interactions within a distance of ≤2 meters. Mote deployment and data retrieval protocols were similar to those previously described [25], [26]. Briefly, motes were placed in a pouch attached to a lanyard, and worn around participants' necks during the school day. Each mote was labeled with a unique identification (ID) number. The beaconing frequency of a mote was 1 per 20 seconds; therefore, data were recorded with a frequency of three recordings per minute. We assume that a potentially contagious contact between two participants occurred if at least one of the two involved motes recorded the other mote's signal. Motes were deployed on three separate days during the spring of 2012. Mote day 1 was Tuesday, January 24th; mote day 2 was Friday, March 2nd; and mote day 3 was Tuesday, March 13th. The weather on each mote day was similar: pleasant and sunny. On the first mote day, paperwork was handed out with the motes that described the study. On those forms, participants could indicate whether they wanted to receive an online health survey by providing an email address. This meant that the online health survey was only sent to individuals who signed up to the study on the first mote deployment day. After registration and entry into the survey website, respondents were sent an email on Saturday, February 4th, 2012, that contained a link to the health survey. Reminder emails were sent four days later before the survey closed on Thursday, February 9th. In addition to demographic questions, the health survey asked participants: “Since August 1, 2011, have you been vaccinated against the flu?” The choice of answer was “Yes” or “No” with horizontal radio buttons next to the choice so that only one answer could be given.

### Network properties

**Figure 1:**
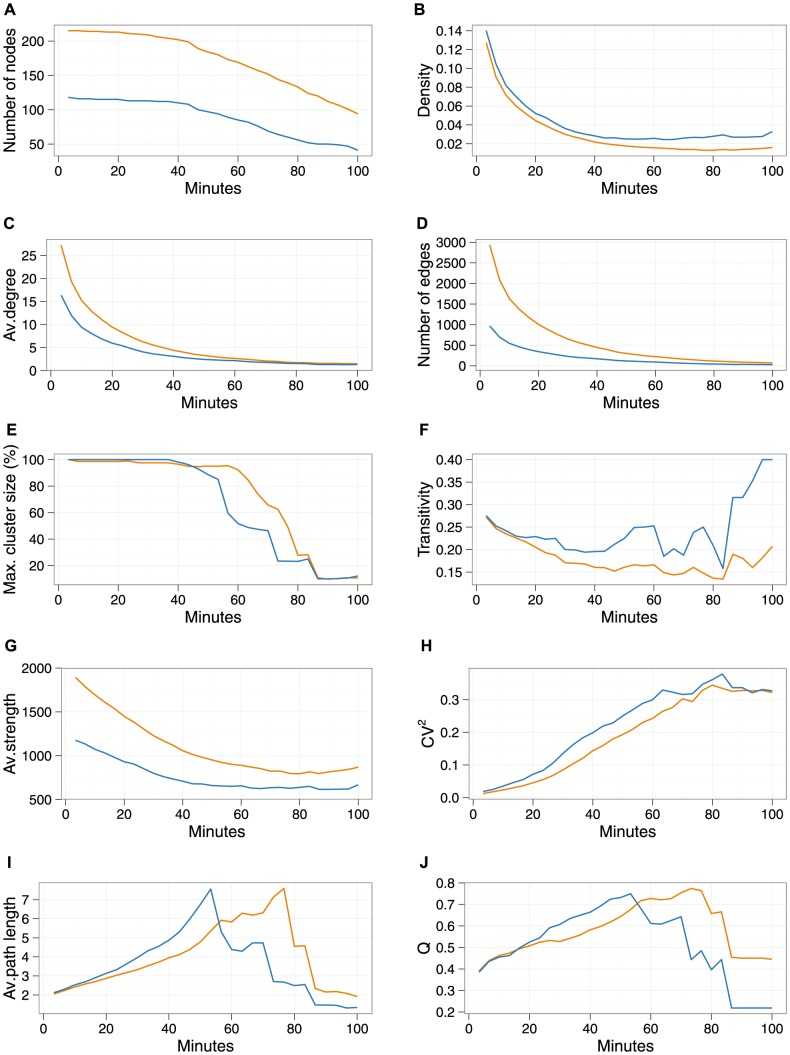
Network statistics on the combined contact data collected during all three school days, for the entire contact graph (orange line) and for the unvaccinated contact graph (blue line). All statistics are calculated for a minimum contact duration (in minutes). As contact duration increases, nodes drop out of the network if they do not have a contact that satisfies the minimum contact duration. (A) Hence, the reduction in the number, V, of nodes. (B) Density of the graph. (C) Average (av.) degree. (D) Number of edges, E. (E) Maximum (max) cluster size, as a fraction of total (maximum) network size. (F) Transitivity (i.e., cluster coefficient). (G) Average (Av.) strength as defined by Barrat [42], where the strength of the node is the total number of CPRs of the node. (H) *CV^2^* of degree. (I) Average (Av.) path length. (J) Modularity, Q, as defined by Reichardt and Bornholdt [53].

**Figure 2:**
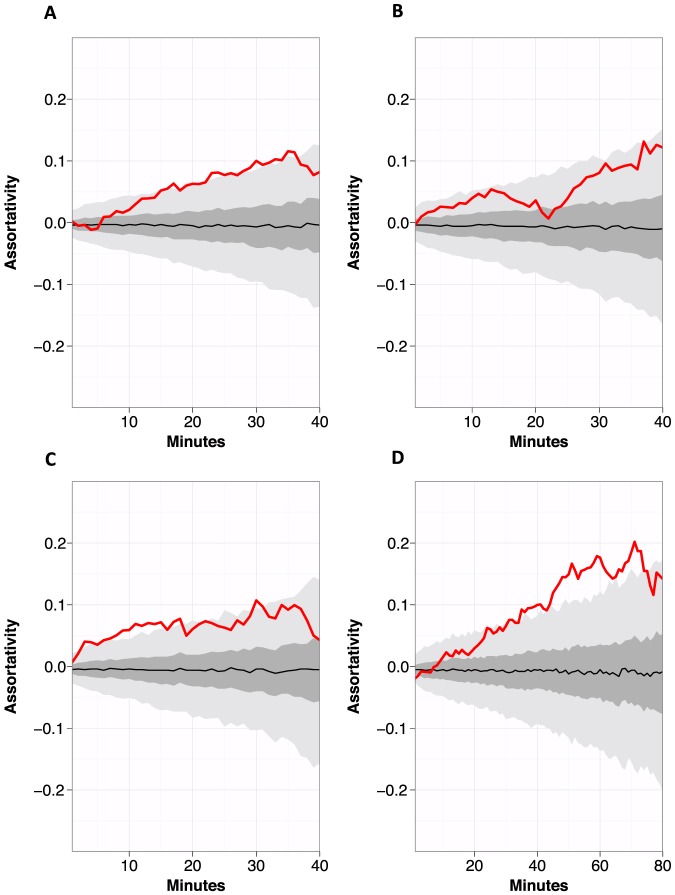
Calculated assortativity coefficient with respect to influenza vaccination status for a minimum contact duration in minutes: (A) on the first day of contact data collection; (B) on the second day of contact data collection; (C) on the third day of contact data collection; (D) on the combined contact data from days 1, 2, and 3. In each panel, the red line represents the assortativity coefficient of the measured network and the black line represents the median assortativity coefficient where vaccination status was randomly allocated to nodes in the network. The dark gray area covers the range from the first to the third quartile of the random networks. The light gray area covers the range from the 2.5 to the 97.5 percentile of the random networks.

Network properties relevant for the spread of infectious disease including number of nodes and edges, density, average degree, maximum cluster size, transitivity, average strength, coefficient of degree variance, average path length, modularity, and vaccination assortativity, were all calculated using igraph 0.6 in R 2.15.1. Vaccination assortativity was calculated using the assortativity coefficient, r (where r>0 describes a positively assortative network, where there is a tendency of nodes to associate with similar nodes with respect to a given property, and r<0 describes a network, where there is a tendency of nodes to associate with dissimilar nodes [19], [20]). Of note, for each individual mote day, or all three mote days combined, the contact duration (in minutes) at which the average path length peaked for the entire network (Fig. 1I; Fig. S1I–S3I) was used as the maximum contact duration for the assortativity plots (Fig. 2), because networks disassemble beyond these values.

### Network assortativity and largest component size

We compared the empirical networks to networks with randomized vaccination patterns with respect to both vaccination assortativity and largest component size. We iterated through a range of contact duration cutoffs with a step width of three CPR (i.e., one minute). For each cutoff, we calculated the vaccination assortativity and the size of the largest connected component of the sub-network of non-vaccinated individuals using both the empirical contact and the self-reported vaccination data. We then created 1,000 different realizations of networks with random vaccination patterns based on the unaltered, empirical contact data and shuffled vaccination data. To create the 1,000 realizations, we picked two random, not isolated nodes and swapped their vaccination status. This procedure was repeated 300 times for each of the 1,000 realizations. Finally, we calculated the vaccination assortativity and the size of the largest connected component of the sub-network of non-vaccinated individuals for each of these 1,000 networks.

### Disease outbreak simulations

We used an individual-based model of influenza spread to elucidate the effect of vaccination assortativity on disease spread. The core of the model is described in detail elsewhere [22], and was implemented in Python 2.7.3 (EPD 7.3-1, 32-bit). Briefly, we assumed that only one randomly selected non-vaccinated individual at the beginning of each simulation run introduces the disease. All further infections happen within the school population and no further cases were introduced from outside. We further assumed that for infection transmission, a minimal cumulative contact duration of 30 minutes (90 CPRs) per simulation time step was required. We used a SEIR-type model with time steps of 12 hours. A simulation week consisted of 14 half days. We assumed that no contacts among school members were made during the half days that cover the nighttime as well as on weekends. Potentially infectious contacts between school members took place during the half days at school.

The probability that a susceptible individual switches to the exposed state per time step was (1−(1−0.00767)*^w^*), where *w* is the accumulated contact time (in CPR) the susceptible individual spent with infected individuals while at school. Exposed individuals became infectious after a period of time which follows a Weibull distribution with an offset of half a day and λ = 1.10 and *k* = 2.21. Due to the rapid deterioration in health associated with infection with influenza, it is unlikely that a sick individual would have contact behaviors similar to a healthy individual. To account for this in our model, we reduced *w* by 75% in the time step during which the individual became infectious, and by 100% in the following time steps before recovery. That means, that infected individuals were confined to their home and, hence, removed from the school population after one time step.

### Contact networks used for the outbreak simulations

We used two kinds of empirical contact data for our simulations: (i) contact data that were collected during the three different school days in spring 2012 and for which we have empirical vaccination data; and (ii) contact data that were previously collected on one school day in 2010 [26]. We do not have empirical vaccination data for the 2010 dataset. The contact data that was collected in 2010, however, covers almost the entire (94%) school population.

We assumed that non-vaccinated individuals were fully susceptible. Vaccinated individuals were either partially or fully immune, depending on the assumed vaccine efficacy (VE). In an idealized scenario of a vaccine that confers perfect immunity, outbreaks can only spread on the sub-networks that are defined by all non-vaccinated individuals and their close-contact interactions. In the case of VE<1, vaccinated individuals can get infected. However, the individuals' infection probability was multiplied with the relative risk RR = 1−VE, and therefore was lower than that of non-vaccinated individuals. The resulting effective transmission probability was, hence, RR•(1−(1−0.00767)*^w^*).

### Simulations based on the 2012 contact and vaccination data

For the 2012 data, simulations used only the data from those individuals who participated in all three contact data collection days, and who additionally reported their seasonal influenza vaccination status (N = 216). Contact data from one of the three data collection days were then randomly allocated to each half-day during the daytime of the five weekdays. We assumed a VE = 1.0, and ran simulations on two classes of contact networks. The first sets of simulations were run using 100 networks with identical topology and identical vaccination patterns based on the collected contact data and the reported vaccination statuses. The second sets of simulations were run using 100 networks with identical topology based on the collected contact data, but with randomized vaccination patterns. We performed 300,000 simulation runs for each of the 100 networks in the two different classes. Disease dynamics were compared according to the mean outbreak size that resulted from the simulation runs. We defined outbreak size as the total number of infected individuals throughout a simulation run minus the index case.

### Simulations based on the 2010 contact data

In 2010, contact data were collected during one school day as reported previously [26]. The data collection covered 94% of the school population, but information about the participants' vaccination status was not collected. Therefore, we created two different kinds of synthetic vaccination data for the 2010 contact data: (i) we randomly assigned a vaccination status to each member of the population, and (ii) we randomly assigned a vaccination status to each member of the population and changed the pattern until a predefined vaccination assortativity was reached. We aimed for a vaccination assortativity r = 0.1 because the empirical vaccination assortativity of all three days in the 2012 collection was approximately 0.1 for contacts with a minimal duration of 90 CPR (Fig. 2). The procedure with which we achieved predefined vaccination assortativity values is described in the online supplementary material.

We compared simulated outbreaks on networks with randomly assigned vaccination patterns to simulated outbreaks on networks with vaccination assortativity r = 0.1 with respect to outbreak probability. Since seasonal influenza vaccine efficacy varies and depends on a number of different factors [27]–[32], and vaccination uptake, among other factors, are subject to behavioral influences [22], [33], we allowed vaccination coverage of 40, 50, and 60 percent and influenza VE values of 0.5, 0.6, 0.7, 0.8, 0.9, and 1.0. For every possible combination of vaccination coverage and vaccine efficacy, we ran 10,000 simulation runs for each of 100 different settings with randomly assigned vaccination patterns, and 10,000 runs for each of 100 different settings with vaccination assortativity r = 0.1.

## Results

### Network structure and vaccination coverage

At the time this project was implemented in 2012, the total school population consisted of 974 individuals (715 students and 259 teachers and staff). We collected CPRs from 564 (58%) individuals of the entire school population on the first day of data collection, 438 (45%) individuals on the second day, and 487 (50%) on the third day. Four hundred and seven (42%) individuals responded to the online health survey. Of these 407 individuals, we obtained contact data for 364 individuals on the first mote day (89.4% of survey respondents), 292 individuals on the second mote day (71.7% of respondents), and 320 individuals on the third mote day (78.6% of respondents).

Overall, from the total of 407 online health survey participants, 169 (41.5%) reported receiving seasonal influenza vaccine; 48.2% of females were vaccinated compared to 33.5% of males (Table S1). According to self-reports, teachers and school staff were better vaccinated (51.9%) than students (39.1%). While the group-specific vaccination coverage differed for the three mote deployment days (Tables S2, S3, S4, S5), the qualitative picture was stable.

We compared network indicators relevant for the spread of infectious disease between the entire network and the sub-network that only contained unvaccinated individuals. Descriptive statistics of these network indicators for all three days combined are shown in Fig. 1, and each of the individual days is shown in Fig. S1, S2, S3. Despite differences in absolute values, all network indicators demonstrated similar trends between the three different data collection days, or when data from all three days were combined. For example, for both networks, a giant component [34] existed until the networks fell apart due to the lack of edges at higher contact durations, the transitivity (ratio of triangles to triads) [34]–[37] was relatively high and the average path length was low, and the coefficient of degree variance squared (*CV^2^*) - relevant because basic reproductive number increases for fixed transmissibility as *CV^2^* increases [38] - was slightly higher with longer contact duration, but overall remained at very low levels. Finally, for both networks, the community structure (modularity) [39] was relatively high, indicating more contact within subgroups than between subgroups. The larger size of the full network was reflected in the greater total number of nodes (individuals), edges (total number of contacts), degree (number of contact partners per node) [40], [41] and strength (degree weighted by duration) [42]. Overall, the structure of both networks was found to be a modular network with small world characteristics and with narrow degree distributions.

### Assortativity of influenza vaccination coverage

The assortativity coefficient r [19], [20], was calculated with respect to the influenza vaccination status on contact data collected on the first, second, and third day of mote deployment, and when contact data from all three of the days were combined. Of significance, we found that unvaccinated individuals tended to associate more often with other unvaccinated than vaccinated individuals, and that this positive assortativity increased (i) with longer contact durations, and (ii) when the data from all three days were combined. Further, the assortativity coefficient, r, of the measured network was above the 97.5^th^ percentile of assortativity coefficient resulting from random vaccination patterns, 28% (Day 1; Fig. 2A), and 43% (Day3; Fig. 2C) of the time on single data collection days, and 50% of the time when data from all three days were combined (Day 123 combined; Fig. 2D). In other words, the assortativity calculated on the measured network was significantly more positive than what would have been expected by chance.

Positive assortativity with respect to the influenza vaccination status across the entire school network could be driven by differences in vaccination coverage and assortativity within sub-networks, such as age, role (student or teacher/staff), gender, or ethnicity. Using data collected from the online health survey, we found differences in vaccination coverage with respect to gender, with more of the female than male population being vaccinated (48.2% versus 33.5% respectively), and with more teachers/staff being vaccinated than students (51.9% and 39.1% respectively) (Table S1). We repeated the above network analyses for each of the three individual days and all three days combined, where we had both contact data and survey data (Table S2, S4, S5). We then calculated the extent to which the different sub-networks contributed to vaccination assortativity and if supposed relationships between demographic variables and vaccination patterns were statistically significant. We found a statistically significant relationship between gender and vaccination patterns, with males contributing more and females contributing less towards assortativity than expected if gender and vaccination were unrelated (Table S6). Statistical significance was determined with a permutation approach. The full protocol is reported in the supplementary material.

### Largest component size

**Figure 3:**
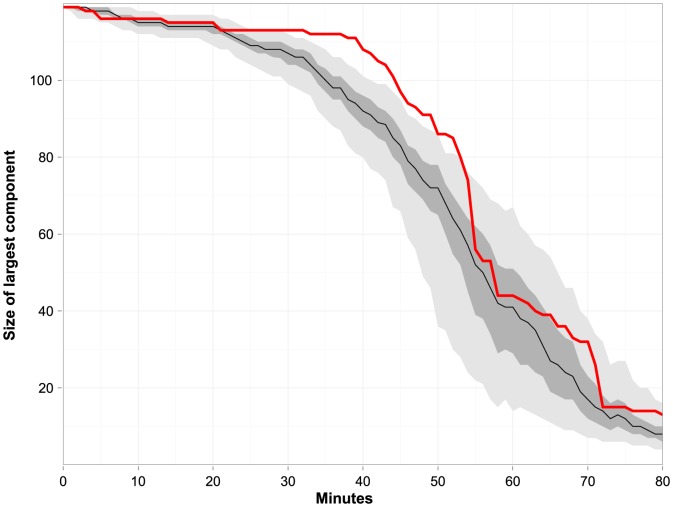
Size of the largest connected component: sub-network of non-vaccinated individuals. The figure is based on the cumulative network of all three data collection days. The red line shows the empirical size of the largest component for a minimum contact duration in minutes. The black line shows the median size of the largest component for identical contact networks with random vaccination patterns for a minimum contact duration; the dark gray area covers the range from the first to the third quartile. The light gray area covers the range from the 2.5 to the 97.5 percentile.

When we calculated the size of the largest component on the measured unvaccinated contact network from all three days, and compared it to an identical contact network where unvaccinated nodes were randomly distributed, we found that for a given contact duration, the size of the largest component was almost always higher for the network measured in this study than for the network with random vaccination patterns (Fig. 3). For contact durations ≤40 min, the measured network essentially consisted of one large component.

### Biases of non-participation on positive vaccine assortativity

Non-participation in both the contact study and the online health survey could have influenced the measured vaccination coverage as well as the observed positive assortativity of vaccination status. Using a larger and almost complete contact data set that we collected from the same school in 2010 [26], we tested whether non-participation could have resulted in biases in our results. In particular, we allowed nodes to drop out of the network (i) randomly or (ii) in positively assortative manner. The full protocol is reported in the supplementary material. Our results indicate that the vaccination coverage of the participating subpopulation is an unbiased estimate of the school-wide vaccination coverage (Fig. S4), and also that the measured assortativity in influenza vaccination is either unbiased (in the case of random non-participation) or may actually underestimate assortativity (in the case of positively assortative non- participation) (Fig. S5). Together, these results suggest that our observation of positive assortativity with respect to influenza vaccination is either not biased or even underestimated by non-participation.

### Simulations for mean outbreak size on positively assortative versus random networks

We simulated influenza outbreaks on the measured, assortative network, and on 100 networks with identical topology, but where the vaccination status of the nodes was randomly rearranged. We assumed that an index case becomes infected outside of school on a random day during the week and disease transmission at the school occurs during half of each weekday. These simulation settings represent a base scenario wherein a single infectious index case introduces the disease into the school population. We found that there was still a 14.9% increase in mean outbreak size on the measured assortative network when we presumed that infected individuals removed themselves from school after 2 hours, assuming an 8-hour school day (Welch's t-test: t (108.2) = 13.3, p<.001), and a 21.2% increase in mean outbreak size when we assumed infected individuals remained at school for the entire day (Welch's t-test: t (104.9) = 15.5, p<.001).

### Simulations for likelihood of large disease outbreaks on positively assortative versus random networks

**Figure 4:**
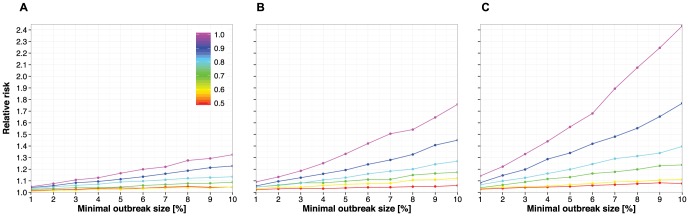
Probability of disease outbreaks that involve at least a given fraction of the susceptible population for contact networks with positively assortative vaccination status relative to contact networks with randomly distributed vaccination status. Networks were constructed by adding a vaccination status to all nodes of the contact network at 90 CPR that was measured at the high school in 2010. Simulations used 100 networks with randomly distributed vaccination status and 100 networks with positively assortative vaccination status (assortativity index r = 0.1 at 90 CPR). Relative risks of outbreaks (vertical axes) are defined as the ratio of the median of the vaccine assortative networks' outbreak probabilities to the median of the outbreak probabilities in random networks and are based on 10,000 simulation runs for each network setting. Minimal outbreak size (horizontal axes) are defined as percent of the susceptible population, which is the number of unvaccinated individuals plus the number of vaccinated individuals times the complement of the assumed vaccine efficacy (1−VE). Thus, each point on the colored lines represents the difference in probability of a disease outbreak based on the ratios between randomly and positively assortative networks for a given minimal outbreak size, and for different vaccine efficacies of 0.5, 0.6, 0.7, 0.8, & 1.0, and assuming: (A) 40%, (B) 50%, and (C) 60% vaccination coverage.

Positive assortativity with respect to influenza vaccination status also has the potential to increase the likelihood of large disease outbreaks. To quantify this effect, we simulated influenza outbreaks on an almost complete network of close contacts that we measured previously at the same high school in 2010. Fig. 4A–C shows that the relative risk of an influenza outbreak can be increased when susceptibility to disease is positively assortative compared to a contact network with randomly distributed vaccination status, and that this relative difference increases with higher vaccination coverage and higher vaccine efficacies.

## Discussion

In a US high school network of close contacts, we observed that unvaccinated individuals tended to socially associate (the network was positively assortative) more often with other unvaccinated individuals than could be expected by chance, and that assortativity was most pronounced when we analyzed contact data collected over multiple days (Fig. 2). In disease simulation models, the mean outbreak size tended to be larger for positively assortative networks than for identical networks where the vaccination status of each individual was randomly allocated. Gender-based differences in vaccine uptake were especially noteworthy, with more females than males being vaccinated (Tables S1, S2, S3, S4, S5), and with unvaccinated males driving the overall positive vaccine assortativity (Table S6). These data suggest that the assortativity of entire networks can be driven by the differences in vaccination uptake within subgroups.

Seasonal influenza vaccination programs rely on an efficacious vaccine [31], [32], [43], and high vaccine coverage [43]–[46]. Targeted vaccination of school-aged children - the main transmitters of influenza - is believed to be particularly important in averting infections to the wider community [12], [47]. It has also been shown that the highest population-wide effect of vaccination campaigns can be achieved, if the reproduction rate *within* pivotal groups like schools can be brought under the local epidemic threshold [23]. There is an increased understanding, however, that the distribution of vaccinated individuals across populations irrespective of high coverage will also significantly affect disease outcomes. If unvaccinated individuals are socially clustered, the probability of large outbreaks is increased due to reductions in herd immunity [18], [22]. Previous studies have reported assortativity in networks relevant for infectious disease spread. For example, contact network data previously collected from the same school as reported in this study [26] described positive assortativity with respect to role (student, teacher, staff). Further, analysis of online social media data has shown that geographic clustering of sentiments towards vaccination can result in increased probability of infection (if those sentiments result in true intentions to vaccinate or not) [22]. Our data, however, provide the first empirical evidence that vaccination against influenza can be positively assortative across a contact network (Fig. 2), and that this has consequences for simulation models of vaccine- preventable disease outbreaks.

Despite differences in absolute values, we found that patterns of relevant network indicators did not differ qualitatively between the full network and the network of unvaccinated susceptible individuals, during each of the individual days of contact data collection, or when contact data from all three days were combined (Fig. 1; Fig. S1, S2, S3). Positive assortativity with regard to influenza vaccination status, however, was a significant feature of this network and increased in a positive direction for individuals who were in contact for the longest, and was larger when data from all three days were combined (Fig. 2).

Gender-based assortativity in schools has been reported to be relevant for the spread of influenza [4] Our finding of gender-based differences in vaccination coverage at this particular school, with more females being vaccinated than males, is also consistent with previous reports [48]. In this study, however, we further demonstrate that gender-based differences in vaccination status can drive overall vaccine assortativity (Fig. S6, S7; Table S6). This suggests that increasing the vaccination coverage of males in this particular network could reduce vaccination assortativity in addition to increasing overall vaccination coverage. This result has important public health implications, because the strategies needed to increase vaccination coverage in males may be different than for females.

If all unvaccinated individuals form one large connected component, then, at least in principle, all unvaccinated individuals could become infected during an outbreak, even if only a single individual introduced the outbreak. If, however, the network falls apart into numerous disconnected components, since we assume only one seed node, then the maximal outbreak size is limited to the size of the largest of these components. When we calculated the size of the largest component on the measured unvaccinated contact network from all three days, and compared it to an identical contact network where unvaccinated nodes were randomly distributed, we found that for a given contact duration, the size of the largest component was almost always higher for the network measured in this study than for the network with random vaccination patterns (Fig. 3). In particular, the size of the largest component of the measured network was significantly larger than the size of the largest component from a network with a random vaccination distribution for contact durations between 29 and 53 minutes. However, given that the effect of assortativity on the largest component was restricted to contacts of longer duration, and the uncertainty in the duration of contact needed for influenza transmission, the relevance of this for disease transmission warrants further investigation.

When we simulated influenza outbreaks on the measured, assortative network, and compared them to networks with identical topology, but where the vaccination status of the nodes was randomly rearranged, we calculated a 14.9% and 21.2% increase in mean outbreak size on the measured assortative network when we assumed infected individuals removed themselves from school after 2 hours, or remained at school for the entire day 8 hour day, respectively.

Additionally, we show that positive assortativity with respect to influenza vaccination can result in a larger outbreak probability than if vaccination was randomly distributed across a network. The relative difference (risk) in outbreak probability between the assortative and random network also increases with outbreak size. Outbreak relative differences further increase with higher vaccination coverage and higher vaccine efficacies (Fig. 4A–C). This means that although overall increases in vaccine efficacy and vaccination coverage (above 0.6 and 40% respectively) could result in an overall reduction in disease as more people are vaccinated, the risk of a disease outbreak could be considerably underestimated at higher efficacies and higher rates, if assortativity of vaccination status is not taken into account. At lower efficacies and lower rates, however, the relative difference between positively assortative and random network becomes less important, as everyone is more susceptible to disease.

There are several limitations in our study. First, participation for each single mote day and for the online health survey did not cover the entire school population. The group of individuals that participated in all three mote collection days consisted of 216 (22%) individuals out of a total school population of 974. This partial participation rate potentially affected our study results in three ways: (i) as only a sub-network of the school population was covered, any outbreak simulation that is solely based on such a sub-network will unavoidably underestimate potential outbreak dynamics; (ii) our overall sample size was small, making it more difficult to distinguish signals from results of pure chance; (iii) our statistic results based on a small sample size with unknown mechanisms of non-participation might have been erroneous or even biased.

We addressed the first point by simulating influenza outbreaks on the measured network, and compared the outputs with a more complete contact network from data that we previously collected at the same high school in 2010 [26]. To address the second point, we generated networks with identical topology, but randomly rearranged the vaccination status of the nodes. We were able to show that the measured network of unvaccinated individuals was significantly more connected, and the full network had significantly higher vaccination assortativity values than networks with random vaccination (Fig. 2). To address the third point, we tested potential sources of bias due to non-participation and found our results to be non-biased, and in particular our assortativity calculations may potentially underestimate the actual assortativity at the school (Fig. S4, S5).

Another limitation is that our influenza simulations relied on assumptions regarding the host-pathogen system and the biology and mechanics of disease transmission. We assumed that influenza transmission requires close-contact [3], [5], [11], [49], and that very short contacts are not sufficient to transmit infection and some accumulation of infectious material during prolonged contacts is required to initiate infection [50]. Although prolonged contacts in age-based contact matrices were found to explain serological patterns (antibody titers by age group) better than shorter contacts [51]–[52], it remains to be established whether assuming a contact duration threshold in simulations is justified. Further, whether our results are applicable to other US schools or communities, or schools in other countries, remains to be determined. Finally, our vaccine assortativity analyses used self-reported vaccination statuses, which were not verified by health records or by a health care provider.

We empirically measured a contact network at a US high school and found the network to be positively assortative with respect to influenza vaccination status, and that positively assortative networks can increase probabilities of disease outbreak. The strength of this study is that vaccination status was obtained directly from individuals within an empirical network that is relevant for influenza transmission. This compares advantageously to electronic communication networks (e.g., Twitter) that do not necessarily reflect contact networks that are needed to transmit infectious disease [22]. By combining high-resolution contact data with survey data, we detected gender-based differences in self-reported influenza vaccination status that contributed significantly to the measured positive vaccine assortativity. These data highlight that researchers should account for assortativity by vaccination status in mathematical models of infectious disease transmission, and that public health officials, in addition to increasing vaccine efficacy and overall vaccination coverage, should recognize that the distribution of vaccinated individuals across populations could also play a role in outbreak size.

## Supporting Information

Figure S1 **Network statistics from the first day of contact data collection.** See Figure 1 for a description of line colors and the network properties analyzed.
(EPS)

Figure S2 **Network statistics from the second day of contact data collection.** See Figure 1 for a description of line colors and the network properties analyzed.
(EPS)

Figure S3 **Network statistics from the third day of contact data collect.** See Figure 1 for a description of line colors and the network properties analyzed.
(EPS)

Figure S4 **Distribution of vaccination coverage in school sub-populations.** The entire school population had a predefined vaccination coverage of 50%. Light gray boxplots show the vaccination coverage of subpopulations that resulted from a dropout of D = 560 individuals out of 761 from the contact network data collected in 2010 (CPR> = 90); dark gray boxplots from a dropout of D = 360 individuals. Dropout occurred either randomly or with dropout assortativity of r = 0.2 or r = 0.4.
(EPS)

Figure S5 **Distribution of vaccination assortativity in school sub-populations.** The entire school population had a predefined vaccination assortativity of r = 0.2. The light gray boxplots show vaccination assortativity values of sub-populations that resulted from a dropout of D = 560 individuals out of 761 from the contact network data collected in 2010 (CPR> = 90); the dark gray boxplots from a dropout of D = 360 individuals. Dropout occurred either randomly or with dropout assortativity of r = 0.2 or r = 0.4.
(EPS)

Figure S6 **Calculated gender assortativity coefficient for a minimum contact duration in minutes, on the first (red), second (blue), and third (green) days of data collection, and when the data from all three days were combined (purple).**
(EPS)

Figure S7 **Calculated vaccination assortativity coefficient within gender subnetworks: (A) on the first day of contact data collection; (B) on the second day of contact data collection; (C) on the third day of contact data collection; (D) on the combined contact data from days 1, 2 and 3.** In each panel, the solid blue line represents the assortativity coefficient for the female population and the solid red line represents the assortativity coefficient for the male population. The dotted lines represent the respective number of edges in each female and male sub-network.
(EPS)

Table S1 Self-reported^*^ seasonal influenza vaccination coverage by demographic group for all survey participants (n = 407).
(DOCX)

Table S2 Self-reported^*^ vaccination coverage by demographic characteristics for mote day 1, Tuesday, January 24^th^, 2012 (n = 287). Inclusion criteria: (i) at least one contact of at least 90 CPR, and (ii) survey participation.
(DOCX)

Table S3 Self-reported^*^ vaccination coverage by demographic characterisitcs for mote day 2, Friday, March 2^nd^, 2012 (n = 227). Inclusion criteria: (i) at least one contact of at least 90 CPR, and (ii) survey participation.
(DOCX)

Table S4 Self-reported^*^ vaccination coverage by demographic characteristics for mote day 3, Tuesday, March 3^rd^, 2012 (n = 247). Inclusion criteria: (i) at least one contact of at least 90 CPR, and (ii) survey participation.
(DOCX)

Table S5 Self-reported^*^ vaccination coverage by demographic characteristics for mote days 1, 2, and 3 combined (n = 209). Inclusion criteria: (i) at least one contact of at least 90 CPR, and (ii) survey participation.
(DOCX)

Table S6 **Statistic π, a measure of the contribution of a demographic characteristic to network assortativity, by demographic characteristic.** The first line of each cell contains the empirical value of π.The second line contains the empirical 95% confidence intervals for π under the assumption that demographic properties and vaccination patterns are unrelated.
(DOCX)

Methods S1 (DOCX)
