# Supplementary material for: Positive Network Assortativity of Influenza Vaccination at a High School: Implications for Outbreak Risk and Herd Immunity
Source: PLoS One. 2014 Feb 5;9(2):e87042. doi: 10.1371/journal.pone.0087042 (PMC3914803; doi:10.1371/journal.pone.0087042)
Supplement: Table S1 — Self-reported* seasonal influenza vaccination coverage by demographic group for all survey participants (n = 407). (DOCX) [file pone.0087042.s008.docx]

|  |  | Vaccinated | Unvaccinated | Vaccination rate |
| --- | --- | --- | --- | --- |
| Total |  | 169 | 238 | 41.5% |
|  |  |  |  |  |
| Gender | Female | 107 | 115 | 48.2% |
|  | Male | 62 | 123 | 33.5% |
|  |  |  |  |  |
| Role | Student | 129 | 201 | 39.1% |
|  | Teacher/Staff | 40 | 37 | 51.9% |
|  |  |  |  |  |
| Age (students) | 13 (1)/14 (73) | 28 | 46 | 37.8% |
|  | 15 | 33 | 52 | 38.8% |
|  | 16 | 35 | 51 | 40.7% |
|  | 17 (69)/ 18 (16) | 33 | 52 | 38.8% |
|  |  |  |  |  |
| Ethnicity | Asian | 95 | 129 | 42.4% |
|  | White | 55 | 66 | 45.5% |
|  | Other | 1 | 9 | 10.0% |
|  | Unknown | 18 | 34 | 34.6% |

* For all tables: The online health survey was distributed on Saturday, February 4^th^, 2012 to the 407 members of the school population who signed up to the study on the first mote day. Response was voluntary. Participants were asked if they had received seasonal influenza vaccination at any time since August 1, 2011.
