# Supplementary material for: Positive Network Assortativity of Influenza Vaccination at a High School: Implications for Outbreak Risk and Herd Immunity
Source: PLoS One. 2014 Feb 5;9(2):e87042. doi: 10.1371/journal.pone.0087042 (PMC3914803; doi:10.1371/journal.pone.0087042)
Supplement: Table S2 — Self-reported* vaccination coverage by demographic characteristics for mote day 1, Tuesday, January 24th, 2012 (n = 287). Inclusion criteria: (i) at least one contact of at least 90 CPR, and (ii) survey participation. (DOCX) [file pone.0087042.s009.docx]

|  |  | Vaccinated | Unvaccinated | Vaccination rate |
| --- | --- | --- | --- | --- |
| Total |  | 115 | 172 | 40.1% |
|  |  |  |  |  |
| Gender | Female | 67 | 81 | 45.3% |
|  | Male | 48 | 91 | 34.5% |
|  |  |  |  |  |
| Role | Student | 97 | 158 | 38.0% |
|  | Teacher/Staff | 18 | 14 | 56.2% |
|  |  |  |  |  |
| Age (students) | 13 (1)/14 (60) | 24 | 37 | 39.3% |
|  | 15 | 24 | 44 | 35.3% |
|  | 16 | 27 | 36 | 42.9% |
|  | 17 (50)/ 18 (13) | 22 | 41 | 34.9% |
|  |  |  |  |  |
| Ethnicity | Asian | 70 | 100 | 41.2% |
|  | White | 31 | 39 | 44.3% |
|  | Other | 0 | 6 | 0.0% |
|  | Unknown | 14 | 27 | 34.1% |
