# Supplementary material for: Positive Network Assortativity of Influenza Vaccination at a High School: Implications for Outbreak Risk and Herd Immunity
Source: PLoS One. 2014 Feb 5;9(2):e87042. doi: 10.1371/journal.pone.0087042 (PMC3914803; doi:10.1371/journal.pone.0087042)
Supplement: Table S3 — Self-reported* vaccination coverage by demographic characterisitcs for mote day 2, Friday, March 2nd, 2012 (n = 227). Inclusion criteria: (i) at least one contact of at least 90 CPR, and (ii) survey participation. (DOCX) [file pone.0087042.s010.docx]

|  |  | Vaccinated | Unvaccinated | Vaccination rate |
| --- | --- | --- | --- | --- |
| Total |  | 98 | 129 | 43.2% |
|  |  |  |  |  |
| Gender | Female | 63 | 55 | 53.4% |
|  | Male | 35 | 74 | 32.1% |
|  |  |  |  |  |
| Role | Student | 89 | 125 | 41.6% |
|  | Teacher/Staff | 9 | 4 | 69.2% |
|  |  |  |  |  |
| Age (students) | 13 (1)/14 (49) | 19 | 31 | 38.0% |
|  | 15 | 23 | 33 | 41.1% |
|  | 16 | 26 | 34 | 43.3% |
|  | 17 (40)/ 18 (8) | 21 | 27 | 43.8% |
|  |  |  |  |  |
| Ethnicity | Asian | 63 | 77 | 45.0% |
|  | White | 23 | 24 | 48.9% |
|  | Other | 0 | 3 | 0.0% |
|  | Unknown | 12 | 25 | 32.4% |
