# Supplementary material for: Positive Network Assortativity of Influenza Vaccination at a High School: Implications for Outbreak Risk and Herd Immunity
Source: PLoS One. 2014 Feb 5;9(2):e87042. doi: 10.1371/journal.pone.0087042 (PMC3914803; doi:10.1371/journal.pone.0087042)
Supplement: Table S4 — Self-reported* vaccination coverage by demographic characteristics for mote day 3, Tuesday, March 3rd, 2012 (n = 247). Inclusion criteria: (i) at least one contact of at least 90 CPR, and (ii) survey participation. (DOCX) [file pone.0087042.s011.docx]

|  |  | Vaccinated | Unvaccinated | Vaccination rate |
| --- | --- | --- | --- | --- |
| Total |  | 105 | 145 | 42.0% |
|  |  |  |  |  |
| Gender | Female | 69 | 72 | 48.9% |
|  | Male | 36 | 73 | 33.0% |
|  |  |  |  |  |
| Role | Student | 89 | 133 | 40.1% |
|  | Teacher/Staff | 16 | 12 | 57.1% |
|  |  |  |  |  |
| Age (students) | 13 (0)/14 (47) | 18 | 29 | 38.3% |
|  | 15 | 22 | 36 | 37.9% |
|  | 16 | 25 | 35 | 41.7% |
|  | 17 (50)/ 18 (7) | 24 | 33 | 42.1% |
|  |  |  |  |  |
| Ethnicity | Asian | 63 | 85 | 42.6% |
|  | White | 28 | 35 | 44.4% |
|  | Other | 1 | 4 | 20.0% |
|  | Unknown | 13 | 21 | 38.2% |
