# Supplementary material for: Positive Network Assortativity of Influenza Vaccination at a High School: Implications for Outbreak Risk and Herd Immunity
Source: PLoS One. 2014 Feb 5;9(2):e87042. doi: 10.1371/journal.pone.0087042 (PMC3914803; doi:10.1371/journal.pone.0087042)
Supplement: Table S5 — Self-reported* vaccination coverage by demographic characteristics for mote days 1, 2, and 3 combined (n = 209). Inclusion criteria: (i) at least one contact of at least 90 CPR, and (ii) survey participation. (DOCX) [file pone.0087042.s012.docx]

|  |  | Vaccinated | Unvaccinated | Vaccination rate |
| --- | --- | --- | --- | --- |
| Total |  | 93 | 116 | 44.5% |
|  |  |  |  |  |
| Gender | Female | 61 | 52 | 54.0% |
|  | Male | 32 | 64 | 33.3% |
|  |  |  |  |  |
| Role | Student | 77 | 106 | 42,1% |
|  | Teacher/Staff | 16 | 10 | 61.5% |
|  |  |  |  |  |
| Age (students) | 13 (0)/14 (32) | 12 | 20 | 37.5% |
|  | 15 | 22 | 29 | 43.1% |
|  | 16 | 24 | 30 | 44.4% |
|  | 17 (40)/ 18 (6) | 19 | 27 | 41.3% |
|  |  |  |  |  |
| Ethnicity | Asian | 55 | 69 | 44.4% |
|  | White | 26 | 25 | 51.0% |
|  | Other | 1 | 3 | 25.0% |
|  | Unknown | 11 | 19 | 36.7% |
