# Supplementary material for: Positive Network Assortativity of Influenza Vaccination at a High School: Implications for Outbreak Risk and Herd Immunity
Source: PLoS One. 2014 Feb 5;9(2):e87042. doi: 10.1371/journal.pone.0087042 (PMC3914803; doi:10.1371/journal.pone.0087042)
Supplement: Table S6 — Statistic π, a measure of the contribution of a demographic characteristic to network assortativity, by demographic characteristic. The first line of each cell contains the empirical value of π.The second line contains the empirical 95% confidence intervals for π under the assumption that demographic properties and vaccination patterns are unrelated. (DOCX) [file pone.0087042.s013.docx]

|  |  | Day 1 | Day 2 | Day 3 | Days 1, 2, 3 |
| --- | --- | --- | --- | --- | --- |
| Gender | Female | **136 ****  [147,196] | **98 ****  [99, 142] | 145  [142, 194] | **176 ****  [211, 285] |
|  | Male | **196 ****  [136, 185] | **134 ****  [90, 133] | 153  [104, 156] | **282 ****  [173, 247] |
|  |  |  |  |  |  |
| Role (Age) | Student (14) | 63  [50, 90] | 53  [34, 68] | 77 *  [37, 77] | 65  [45, 98] |
|  | Student (15) | 93  [59, 99] | **81 ****  [40, 76] | 91  [48, 92] | 138  [81, 144] |
|  | Student (16) | 82  [53, 93] | 59  [43, 81] | 72  [50, 95] | 143  [87, 151] |
|  | Student (17) | 57  [40, 77] | 26 *  [26, 57] | **33 ****  [40, 81] | 81  [59, 118] |
|  | Teacher/Staff | **15 ****  [23, 53] | 5 *  [5, 24] | **14 ****  [18, 51] | **16 ****  [33, 82] |
|  |  |  |  |  |  |
| Ethnicity | Asian | 206  [172, 220] | 144  [123, 164] | 193  [151, 202] | 297  [235, 307] |
|  | White | **59 ****  [61, 102] | 41  [32, 66] | 56  [54, 98] | **80 ****  [81, 144] |
|  | Other | 6  [1, 15] | 6  [0, 9] | 5  [0, 15] | 10  [0, 20] |
|  | Unknown | 61  [31, 65] | 41  [23, 54] | 44  [24, 59] | 71  [41, 92] |

** clearly significant with < .05

* borderline significant with p = .05
