# Supplementary material for: Positive Network Assortativity of Influenza Vaccination at a High School: Implications for Outbreak Risk and Herd Immunity
Source: PLoS One. 2014 Feb 5;9(2):e87042. doi: 10.1371/journal.pone.0087042 (PMC3914803; doi:10.1371/journal.pone.0087042)
Supplement: Methods S1 — (DOCX) [file pone.0087042.s014.docx]

S1 Methods

For article Barclay et al. Positive network assortativity of influenza vaccination at a high school: implications for outbreak risk and herd immunity

Bias testing

The positive assortativity in vaccination status that we observed could have been influenced by the participation rate in the contact study and the number of responses we received to the online health survey. To account for any bias in the data, we tested how various mechanisms of participant dropout might affect vaccination coverage and assortativity. In particular, we analyzed the effect of the following dropout mechanisms (i) Random dropout: where we assume that the decision of a school member not to participate in the study was not correlated with any other property that is included in our analyses (i.e., network position of the individual or its vaccination status). Consequently, the dropout appears to be random; (ii) Positively assortative dropout: where we assume that an individual’s decision to participate in the study is positively correlated with the decisions of the individual’s contact partners: pairs of individuals that recorded at least 90 CPR per mote day were more likely to make the same dropout decision (either positive or negative) than under the random dropout assumption. Positively assortative dropout could be caused by peer influence as well as by homophily during network formation [1-3]. We tested dropout assortativity values of r = 0.2 and r = 0.4.

To simulate and analyze the effects of these dropout mechanisms on vaccination coverage and assortativity, we used a close-contact dataset collected previously from the high school in 2010 [4,5]. We filtered the contacts of the 789 individuals that participated in the 2010 study for contacts of at least 90 CPR. The filtered network consisted of 761 nodes and 7992 edges. A vaccination status (1=vaccinated; 0=susceptible) and a dropout decision (1=dropout; 0=participation) were randomly attributed to each of the 761 nodes so that finally V individuals were vaccinated and D individuals did not participate in the study. We assumed that V = 380 for all simulations. A vaccination assortativity of 0.2 was assumed for the entire population of 761 nodes. To approximate that value, we consecutively swapped the vaccination status of 10^4^ pairs of randomly chosen nodes. For each swap, we tested whether the swap brought the actual vaccination assortativity of the network closer to the target value of 0.2. The swap was reversed if the vaccination assortativity was not brought closer to the target value than before. As a final step, we removed all nodes that were flagged as dropouts and calculated vaccination coverage and vaccination assortativity of the remaining network. These simulations were repeated 200 times for each combination of the following parameters: dropout assortativity r = 0.2 or r = 0.4 as well as random dropout and D = 560 or D = 360.

We found that the empirical vaccination coverage of our sample was an unbiased estimate of the entire school population’s vaccination coverage regardless of whether dropout was random or assortative (Fig. S4). Moreover, the empirical vaccination assortativity appeared to be an unbiased estimate of the school population’s vaccination assortativity, if dropout was random. If drop out was assortative, there would be a tendency to underestimate the real vaccination assortativity (Fig. S5).

Demographic groups: vaccination coverage and contributions to vaccination assortativity

The school population can be subdivided into demographic groups by gender, role, age, or ethnicity. It is possible that each of these sub-networks contribute differently to the overall vaccination coverage, and also to the vaccination assortativity that we measured on the entire school network. In Table S1, we report the vaccination coverage for each of the demographic groups gathered from the online health survey. We also report vaccination coverage for each of the demographic groups, but only from participants who completed the online survey and participated in the first mote day (Table S2), completed the online health survey and participated in the second mote day (Table S3), completed the online health survey and participated in the third mote day (Table S4), or completed the online health survey and participated in all three mote days combined (Table S5). Further, Tables S2-S5 only includes individuals who had at least one contact of at least 90 CPR. Hence, it is possible that a vaccinated individual, who was below that level for an individual day, was above that level for all three days combined. At the same time, a non-vaccinated might have dropped out because she/he did not participate in all three days.

Vaccination coverage of all online health survey participants was 41.5%. We found that there was a 14.7% difference in vaccination coverage by gender (48.2% of females were vaccinated compared to 33.5% of males). There was also a difference in the vaccination coverage of teachers and staff (51.9%) compared to that of students (39.1%). Within the student body, there are only minuscule age differences (that could actually be a result of sampling), and the age-specific vaccination coverage ranged from 37.8% to 40.7%. Further, the difference between the two large ethnic groups, Asians and whites, was small with an absolute value of 3.1%. Members of other ethnic groups reported substantially lower vaccination coverage (10.0%), but only 10 out of 407 individuals reported to belong to an ethnic group other than Asian or white. The vaccination coverage of those individuals who did not reveal their ethnicity (52 individuals) was 34.6%. While group-specific vaccination coverage differed for the three mote deployment days (Tables S2-S5), the qualitative picture was stable.

Positive vaccination assortativity results in disproportionately high numbers of contacts among unvaccinated members of a population. Two unvaccinated individuals who have contact with each other form an unvaccinated dyad. To test whether some demographic groups contributed significantly more or less to the positive vaccination assortativity measured on the entire network, we defined a statistic π as a measure of the contribution of a demographic group to the formation of unvaccinated dyads in the entire population’s contact network.

The statistic π for a particular demographic group is defined as a weighted dyad count: In order to calculate π, we looped over all unvaccinated dyads. For every unvaccinated dyad, π was increased by 1 if exactly one of the two individuals that form the unvaccinated dyad belongs to the analyzed demographic group, and by 2 if both individuals belong to this group. In general, it can be expected that large demographic groups are strongly involved in the formation of unvaccinated dyads whereas the contribution of smaller groups is minor.

We postulate a null hypothesis, H_0_, that demographic groups have no relationship with vaccination patterns and, hence, their involvement in the formation of unvaccinated dyads corresponds solely to the group size. H_0_ is rejected if the proportion of individuals of a specific demographic group in the population’s unvaccinated dyads is significantly lower or higher than would be expected based on the group size (H_1_). To test this hypothesis, we related for each analyzed demographic group the empirical π to the distribution of π values that would be observed when the network and the vaccination distribution are kept constant (i.e., the concrete nodes who are vaccinated stay the same), but the demographic properties of the individuals are randomly redistributed within the population.

As before, we used contact networks of at least 90 CPR. We calculated the statistic for days 1, 2, and 3 separately as well as for days 1, 2, and 3 combined. We created 10,000 populations with randomly redistributed demographic characteristics (permutations); therefore, we swapped the demographics of 1,000 pairs of individuals for each demographic property separately. With the contact networks, the known vaccination patterns, and the 10,000 populations with randomly redistributed demographic characteristics, we can determine a simulated 95% confidence interval for π if demographic properties and vaccination patterns are unrelated defined by the 2.5 percentile and the 97.5 percentile of the simulated π distribution. If the empirical π value was not included in the 95% confidence interval, it was considered to be significantly different from a random configuration on an alpha level of 0.05. The applied permutation methodology is similar to permutation approaches used in previous studies [6-10].

Table S6 shows the empirical π values as well as the simulated 95% confidence intervals to test H_0_ for all four contact networks. Females contributed significantly less than expected and males significantly more than expected to the vaccination assortativity at day 1, day 2, and days 1, 2, and 3 combined. The simulated confidence intervals for day 3 contained the empirical π values, and, hence, the empirical π values were not statistically different from the simulation outcomes on a level of 5 percent, but the trend is congruent with the findings from the other mote days. Based on these findings, H_0_ is rejected for gender; instead the alternative hypothesis H_1_ has to be chosen, which says that the two genders contribute differently to vaccination assortativity. Additionally, teachers and staff contributed significantly less to unvaccinated dyads as they would, if vaccination and demography were unrelated.

Fig. S6 and S7 show the calculated network assortativity coefficient for gender, and gender in relation to influenza vaccination status, respectively. There was gender assortativity; males tended to socially associate more with other males than with females, and vice versa (Fig. S6). Assortativity with respect to influenza vaccination within gender subnetworks was low, and only increased when few edges remained in the subnetworks (Fig. S7).

S1 **References**

- 1. Lewis K, Gonzalez M, Kaufman J (2011) Social selection and peer influence in an online social network. Proc Natl Acad Sci U S A 109:68-72. doi: 10.1073/pnas.1109739109

2. CM, Valente TW (2012) Social integration in friendship networks: the synergy of network structure and peer influence in relation to cigarette smoking among high risk adolescents. Soc Sci Med 74: 1407–1417. doi: 10.1016/j.socscimed.2012.01.011

3. Hunter SM, Vizelberg IA, Berenson GS (1991) Identifying mechanisms of adoption of tobacco and alcohol use among youth: The Bogalusa heart study. Soc Net 13: 91–104. .doi:10.1016/0378-8733(91)90015-L

4. Kazandjieva MA, Lee JW, Salathé M, Feldman MW, Jones JH, et al. (2010) Experiences in measuring a human contact network for epidemiology research. Proceedings of the 6th workshop on hot topics in embedded network sensors. Article No. 7

5. Salathé M, Kazandjieva M, Lee JW, Levis P, Feldman MW, et al. (2010) A high-resolution human contact network for infectious disease transmission. Proc Natl Acad Sci U S A 107: 22020–22025. doi:10.1073/pnas.1009094108

6. Emelianov I, Drès M, Baltensweiler W, Mallet J (2001) Host-induced assortative mating in host races of the larch budmoth. Evolution 55: 2002–2010. doi:10.1111/j.0014-3820.2001.tb01317.x

7. Salathé M, Khandelwal S (2011) Assessing vaccination sentiments with online social media: implications for infectious disease dynamics and control. PLoS Comput Biol 7: e1002199. doi:10.1371/journal.pcbi.1002199

8. Smieszek T, Salathé M (2013) A low-cost method to assess the epidemiological importance of individuals in controlling infectious disease outbreaks. BMC Med 11: 35. doi: 10.1186/1741-7015-11-35

9. Wang J, Suri S, Watts DJ (2012) Cooperation and assortativity with dynamic partner updating. PNAS 109: 14363–14368. doi:10.1073/pnas.1120867109

10. Bliss CA, Kloumann IM, Harris KD, Danforth CM, Dodds PS (2012) Twitter reciprocal reply networks exhibit assortativity with respect to happiness. J Comput Sci 3: 388–397. doi:10.1016/j.jocs.2012.05.001
